# Effects of danofloxacin dosing regimen on gastrointestinal pharmacokinetics and fecal microbiome in steers

**DOI:** 10.1038/s41598-021-90647-z

**Published:** 2021-05-27

**Authors:** J. L. Halleran, B. J. Callahan, M. E. Jacob, H. J. Sylvester, T. Prange, M. G. Papich, D. M. Foster

**Affiliations:** 1 Department of Population Health and Pathobiology, College of Veterinary Medicine, NC State University, Raleigh, NC USA; 2 Department of Clinical Sciences, College of Veterinary Medicine, NC State University, Raleigh, NC USA; 3 Deparment of Biomedical Sciences, College of Veterinary Medicine, NC State University, Raleigh, NC USA

**Keywords:** Molecular biology, Gastroenterology

## Abstract

Fluoroquinolones are a class of antimicrobial commonly used in human medicine, and deemed critical by the World Health Organization. Nonetheless, two formulations are approved for the treatment of respiratory disease in beef cattle. The objective of this study was to determine the gastrointestinal pharmacokinetics and impact on enteric bacteria of cattle when receiving one of the two dosing regimens (high: 40 mg/kg SC once or low: 20 mg/kg IM q48hr) of danofloxacin, a commonly utilized synthetic fluoroquinolone in veterinary medicine. Danofloxacin was administered to 12 steers (age 7 months) fitted with intestinal ultrafiltration devices at two different dosing regimens to assess the gastrointestinal pharmacokinetics, the shifts in the gastrointestinal microbiome and the development of resistant bacterial isolates. Our results demonstrated high intestinal penetration of danofloxacin for both dosing groups, as well as, significant differences in MIC values for *E. coli* and *Enterococcus* between dosing groups at selected time points over a 38 day period. Danofloxacin treatment consistently resulted in the *Euryarchaeota* phyla decreasing over time, specifically due to a decrease in *Methanobrevibacter*. Although microbiome differences were minor between dosing groups, the low dose group had a higher number of isolates with MIC values high enough to cause clinically relevant resistance. This information would help guide veterinarians as to appropriate dosing schemes to minimize the spread of antimicrobial resistance.

## Introduction

Fluoroquinolones are considered a critically important antibiotic in human medicine^1,2^. Therefore, in the United States and European Union, the use of fluoroquinolones in veterinary medicine is restricted to on label usage as there are concerns that increased use can lead to an increased risk of antimicrobial resistance (AMR) in both veterinary and human patients^3,4^. Fluoroquinolone bacterial resistance is associated with three recognized mechanisms: mutations that alter the target of the drug, mutations that will reduce drug accumulation, and the presence of plasmids that encode genes that protect the cell^5,6^. Genes associated with fluoroquinolone resistance have been documented in cattle feces through metagenomics studies, demonstrating that feces could be a source of AMR transmission to water or crops^7–9^. Dosing regimen has been found to play a role in the development and acquisition of AMR in bacteria. “Inadequate exposure” of a therapeutic occurs when a sub-therapeutic administration of an antibiotic is given, resulting in inefficient killing of bacteria with borderline susceptibility^10^. These borderline bacteria will survive, leading to an antibiotic resistant population. Therefore, dosing regimens should be designed to achieve adequate therapeutic drug concentrations early in treatment to eliminate the borderline susceptible group of bacteria in order to decrease risk of resistant populations.

Danofloxacin, a synthetic fluoroquinolone, is a commonly used veterinary antimicrobial currently labeled for treatment and control of bovine respiratory disease associated with *Mannheimia haemolytica* and *Pasteurella multocidia* in beef cattle^1^. Like other fluoroqinolones, danofloxacin acts by inhibiting bacterial DNA gyrase, preventing DNA replication, causing it to be bactericidial. Danofloxacin’s veterinary label has two dosing regimens, a lower multi-day therapy and higher single dose therapy. The withdrawal time required prior to an animal entering the food chain after treatment with either regimen is 4 days after the last dose. This short withdrawal time is beneficial to owners, but may allow for the persistence of resistant bacteria when the animal goes to slaughter. This antimicrobial is marketed for treatment of respiratory disease, yet administration of parenteral antibiotics can affect the composition and susceptibility of the gastrointestinal microbiota^10–13^. Understanding the gastrointestinal pharmacokinetic/pharmacodynamic relationship and how it correlates to minimum inhibitory concentrations (MICs) and microbiome changes can shed light on the development of AMR, providing guidance for clinical use of these drugs.

The overall objective of this study was to assess the pharmacodynamic properties associated with both dosing regimens of danofloxacin and the subsequent development of antimicrobial resistance in enteric bacteria.

We have a two-part hypothesis. First, steers administered the lower dose of danofloxacin twice will have lower gastrointestinal concentrations when compared to the steers whom received the single, high dose of danofloxacin. Second, the decreased gastrointestinal concentrations from the low dose group will allow for an increased development, amplification and persistence of a resistant sub-population of enteric bacteria. Briefly, this was carried out through dosing at time point 0 with sample collection of blood, intestinal ultrafiltrate and interstitial fluid at various time points over the first 7 days of the study. Feces was collected over 27 days, allowing for a longitudinal design to assess changes in antimicrobial resistance over time.

## Results

### Pharmacokinetic modeling

**Figure 1:**
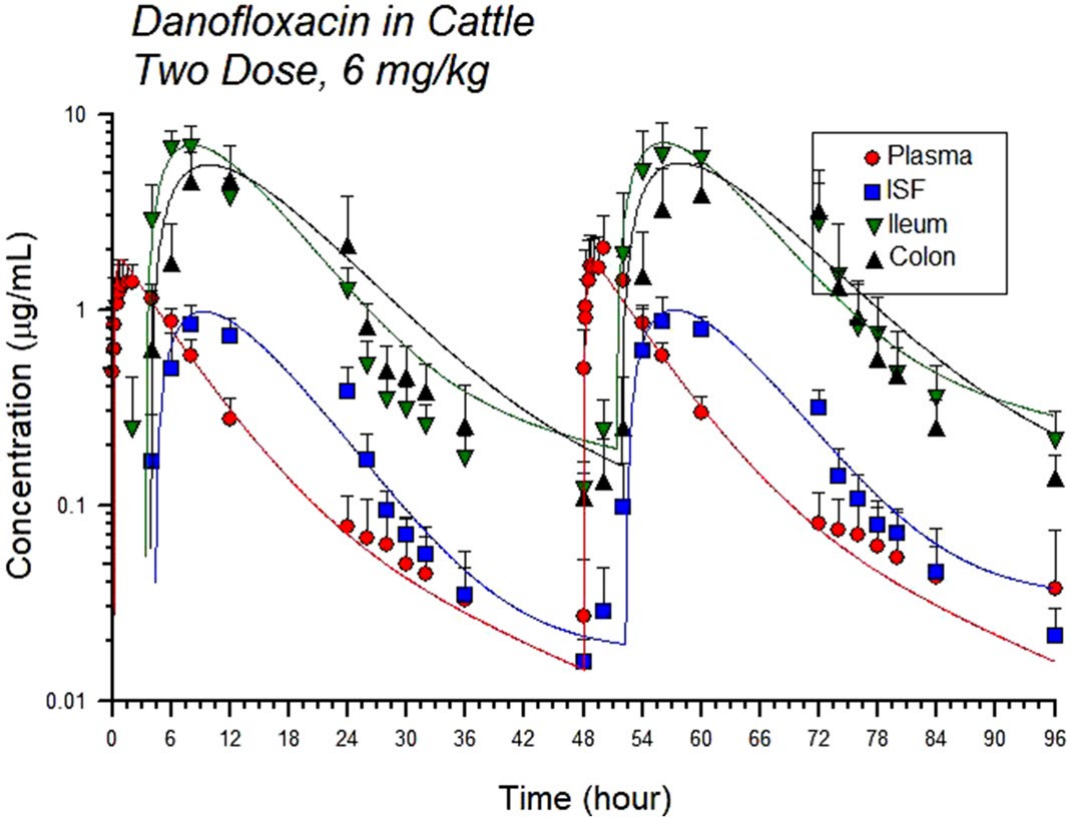
Total concentration of danofloxacin in plasma, interstitial fluid (ISF), ileum and colon ultrafiltrate dosed at 6 mg/kg, once every 48 h*.* Each point represents the mean and the error bars represent the standard deviation. The circle indicates plasma concentrations, the square indicates ISF concentrations, the upside down triangle indicates ileum ultrafiltrate concentrations and the right side up triangle indicates colon ultrafiltrate concentrations. The data is presented on a semi-logarithmic axis. The solid lines represent the predicted concentration based on the model fit. The actual values are the solid points.

**Figure 2:**
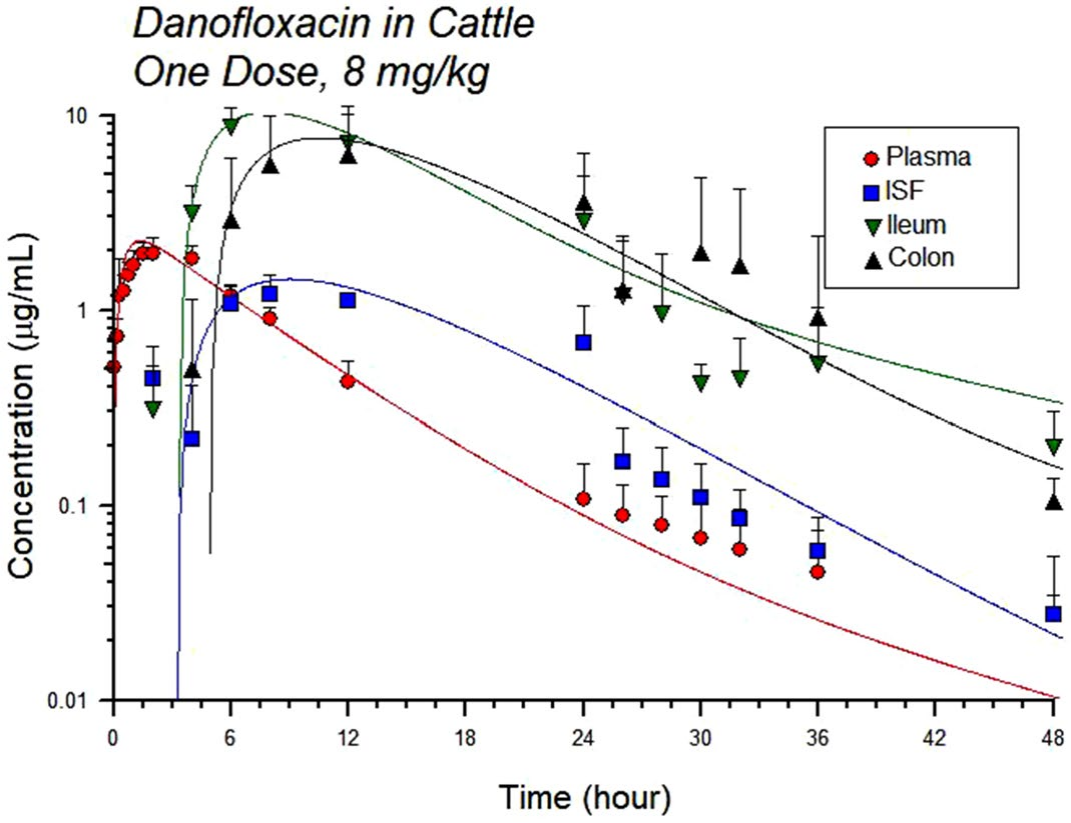
Total concentration of danofloxacin in plasma, interstitial fluid (ISF), ileum and colon ultrafiltrate dosed at 8 mg/kg once*.* Each point represents the mean and the error bars represent the standard deviation. The circle indicates plasma concentrations, the square indicates ISF concentrations, the upside down triangle indicates ileum ultrafiltrate concentrations and the right side up triangle indicates colon ultrafiltrate concentrations. The data is presented on a semi-logarithmic axis. The solid lines represent the predicted concentration based on the model fit. The actual values are the solid points.

The values of the pharmacokinetic parameters for both dosing groups in all sample types are shown in Tables 1 and 2. The mean AUC for the low group was 14.08 h ug/ml and for the high dose group was 19.39 h ug/ml. The mean plasma half-life was approximately 20 h from both doses (Figs. 1, 2). The half-life for interstitial fluid was also significantly different between dosing groups (p value < 0.05); it was higher in the low dose group. The maximum drug concentration was significantly different between the dosing groups in the ileum gastrointestinal ultrafiltrate (p value < 0.05); the maximum drug concentration was increased in the high dose group. All other parameters were not significantly different between dosing groups for plasma, interstitial fluid, ileum or colon ultrafiltrate. There was high intestinal penetration of approximately 700% or higher in the ileum and 500–600% in the colon following administration of both the high and low doses of danofloxacin (Tables 1, 2, 3, 4, Figs. 1, 2).

**Table 1:** Pharmacokinetic parameters for danofloxacin in plasma dosed at 6 mg/kg under the skin every 48 h for two doses and 8 mg/kg under the skin once.

Parameter	Units	6 mg/kg		8 mg/kg	
		Geometric mean	Geometric CV %	Geometric mean	Geometric CV %
A	ug/ml	1.938	27.790	2.847	25.566
Alpha	1/h	0.181	12.073	0.167	18.365
Alpha t½	h	3.833	11.996	4.154	18.389
AUC	h ug/ml	13.647	27.970	19.179	16.258
B	ug/ml	0.125	67.867	0.167	45.863
Beta	1/h	0.043	37.142	0.046	94.110
Beta t½	h	17.587	52.043	15.015	92.243
CMAX	ug/ml	1.625	26.801	2.002	16.936
K01	1/h	3.409	102.592	1.734	66.131
K01 t½	h	0.204	102.389	0.400	66.157
TMAX	h	0.910	67.713	1.479	39.938

A, B, the intercept for the initial and terminal portions of the curve, respectively. Alpha (α) and beta (β) are the rate constants for the initial and terminal portions of the curves, with their respective half-lives (t½). K01 is the absorption rate and corresponding half-life (t½).

*C*_*MAX*_ maximum drug concentration, *T*_*MAX*_ time to maximum drug concentration.

**Table 2:** Pharmacokinetic parameters for danofloxacin in interstitial fluid (ISF) dosed at 6 mg/kg under the skin every 48 h for two doses and 8 mg/kg under the skin once.

Parameter	Units	6 mg/kg		8 mg/kg	
		Geometric mean	Geometric CV %	Geometric mean	Geometric CV %
A	ug/ml	17.880	15.777	8.806	130.186
Alpha	1/h	0.188	8.701	0.148	14.864
Alpha t½	h	3.681	8.817	4.695	14.924
AUC	h ug/ml	21.915	36.333	18.102	12.778
B	ug/ml	0.018	55.676	0.099	126.681
Beta	1/h	0.002	36.677	0.036	227.671
Beta t½	h	341.702	21.675	11.289	105.570
CMAX	ug/ml	0.934	20.799	1.013	23.908
K01	1/h	0.217	10.821	0.198	31.149
K01 t½	h	3.189	10.882	3.501	31.256
TMAX	h	9.243	14.389	8.175	16.586
Penetration	%	160.588	20.644	94.388	26.063

A, B, the intercept for the initial and terminal portions of the curve, respectively. Alpha (α) and beta (β) are the rate constants for the initial and terminal portions of the curves, with their respective half-lives (t½). K01 is the absorption rate and corresponding half-life (t½).

*C*_*MAX*_ maximum drug concentration, *T*_*MAX*_ time to maximum drug concentration.

**Table 3:** Pharmacokinetic parameters for danofloxacin in ileum ultrafiltrate dosed at 6 mg/kg under the skin every 48 h for two doses and 8 mg/kg under the skin once.

Parameter	Units	6 mg/kg		8 mg/kg	
		Geometric mean	Geometric CV %	Geometric mean	Geometric CV %
A	ug/ml	308.750	6.384	67.993	54.832
Alpha	1/h	0.207	12.018	0.199	12.708
Alpha t½	h	3.347	12.098	3.481	12.660
AUC	h ug/ml	104.030	21.868	137.140	41.004
B	ug/ml	0.425	76.988	0.889	326.893
Beta	1/h	0.020	46.706	0.038	77.956
Beta t½	h	34.820	46.666	18.395	77.212
CMAX	ug/ml	6.889	13.081	9.083	23.704
K01	1/h	0.220	11.733	0.274	13.407
K01 t½	h	3.156	11.809	2.528	13.474
TMAX	h	8.123	14.836	7.683	4.113
Penetration	%	762.313	14.829	715.072	30.575

A, B, the intercept for the initial and terminal portions of the curve, respectively. Alpha (α) and beta (β) are the rate constants for the initial and terminal portions of the curves, with their respective half-lives (t½). K01 is the absorption rate and corresponding half-life (t½).

*C*_*MAX*_ maximum drug concentration, *T*_*MAX*_ time to maximum drug concentration.

**Table 4:** Pharmacokinetic parameters for danofloxacin in spiral colon ultrafiltrate dosed at 6 mg/kg under the skin every 48 h for two doses and 8 mg/kg under the skin once.

Parameter	Units	6 mg/kg		8 mg/kg	
		Geometric mean	Geometric CV %	Geometric mean	Geometric CV %
A	ug/ml	331.907	9.860	40.642	486.117
Alpha	1/h	0.168	10.235	0.155	22.222
Alpha t½	h	4.115	10.262	4.457	22.125
AUC	h ug/ml	84.318	45.630	106.836	179.445
B	ug/ml	0.343	140.751	0.164	84.429
Beta	1/h	0.030	45.670	0.016	109.624
Beta t½	h	23.318	46.105	42.468	106.580
C_MAX_	ug/ml	4.851	49.544	4.564	182.458
K01	1/h	0.176	11.203	0.197	25.205
K01 t½	h	3.947	11.135	4.438	87.278
T_MAX_	h	9.800	6.770	12.902	51.385
Penetration	%	596.575	59.041	579.725	220.847

A, B, the intercept for the initial and terminal portions of the curve, respectively. Alpha (α) and beta (β) are the rate constants for the initial and terminal portions of the curves, with their respective half-lives (t½). K01 is the absorption rate and corresponding half-life (t½).

*C*_*MAX*_ maximum drug concentration, *T*_*MAX*_ time to maximum drug concentration.

### Plasma protein binding

The plasma protein binding was 36.4% (0.37 Std.Dev) for the concentration of 1.0 µg/ml, and 39.4% (0.31 standard deviation) for the concentration of 0.5 µg/ml.

### Concentration of *E. coli* and *Enterococcus*

**Figure 3:**
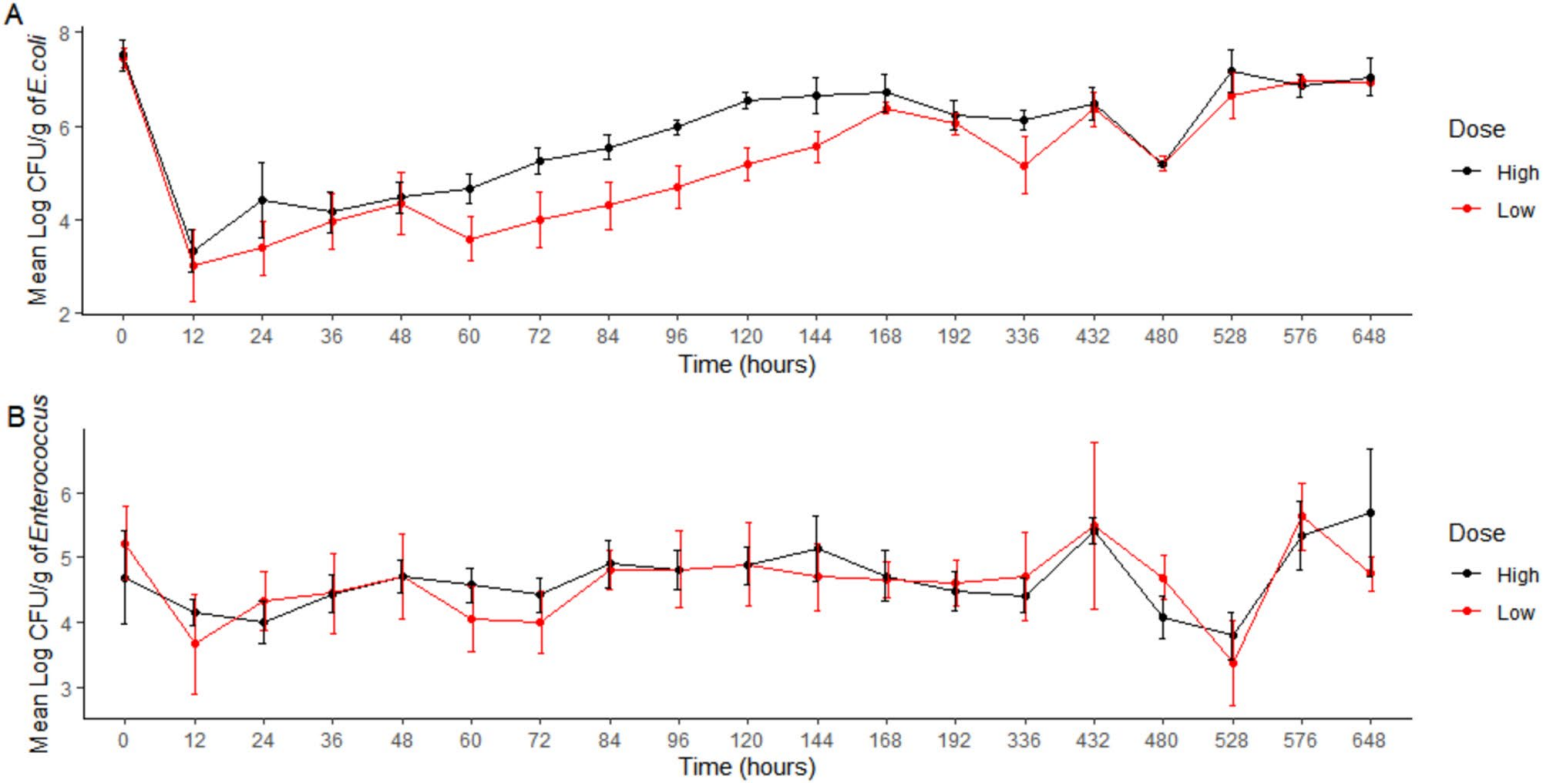
Mean Log CFU/g of *E. coli* and Enterococcus per dosing group over time with standard error. (**A**) Shows the Mean log CFU/g of *E.* coli while (**B**) shows the mean log CFU/g of Enterococcus. The black line is the high dose group and the red line is the low dose group. Individual t-tests with a Bonferonni correction (p < 0.0125) were performed for overall log CFU/g at predetermined time points. The only significant difference seen for *E. coli* was comparing Day 0 to Day 6 for the low dose group only (p = 0.0006). There was no significant differences noted for *Enterococcus*.

Figure 3 shows the mean concentration (log_10_CFU/g) over time per dosing group for both *E. coli* (3A) and *Enterococcus* (3B) respectively. The concentration of *E. coli* dropped substantially after administration of danofloxacin, but it recovered and returned to baseline over time. There was significant difference in log CFU/g of *E. coli* when comparing Day 6 (144 h) to Day 0 for the low dose group (p = 0.0006). There was no significant difference in log CFU/g of *E. coli* when comparing Day 0 to Day 4 (96 h) for the high dose group, or any difference between dosing groups at time point Day 4 and Day 6. These times were specifically evaluated as this is the earliest that the animal could enter the food chain. After administration of danofloxacin, there was no substantial effect on the concentration of *Enterococcus*, with no significant differences observed in log CFU/g between days 0, 4 and 6.

### *E. coli* and *Enterococcus* minimum inhibitory concentration

Descriptive statistics for MIC values for both *E. coli* and *Enterococcus* are shown in Supplementary Tables S1 and S2.

For *Enterococcus* spp*.*, the MIC median spiked for each dosing group after 96 h. Although the medians per group were different, they remained above the zero time point for the rest of the study. Individual predetermined Wilcoxon-ranked sum tests were conducted. There was a significant difference between MIC values for *E. coli* at time point 0 compared to Day 6 (144 h) for the low dose group (Wilcoxon-ranked sum test, p = 0.0005) and at time point 0 compared to Day 4 (96 h) for the high dose group (Wilcoxon-ranked sum test, p = 0.006). In addition, at Day 4 (96 h), there was a significant difference in MIC values for *E. coli* between the dosing groups (Wilcoxon-ranked sum test, p = 3.4e6) and at Day 6 (144 h, Wilcoxon-ranked sum test, p = 0.00025). The MIC values were significantly higher in the low dose group when compared at Day 4 and Day 6. For *Enterococcus*, the only significant difference in MIC values was noted when comparing time point 0 to Day 6 for the low dose group (p = 0.006).

**Figure 4:**
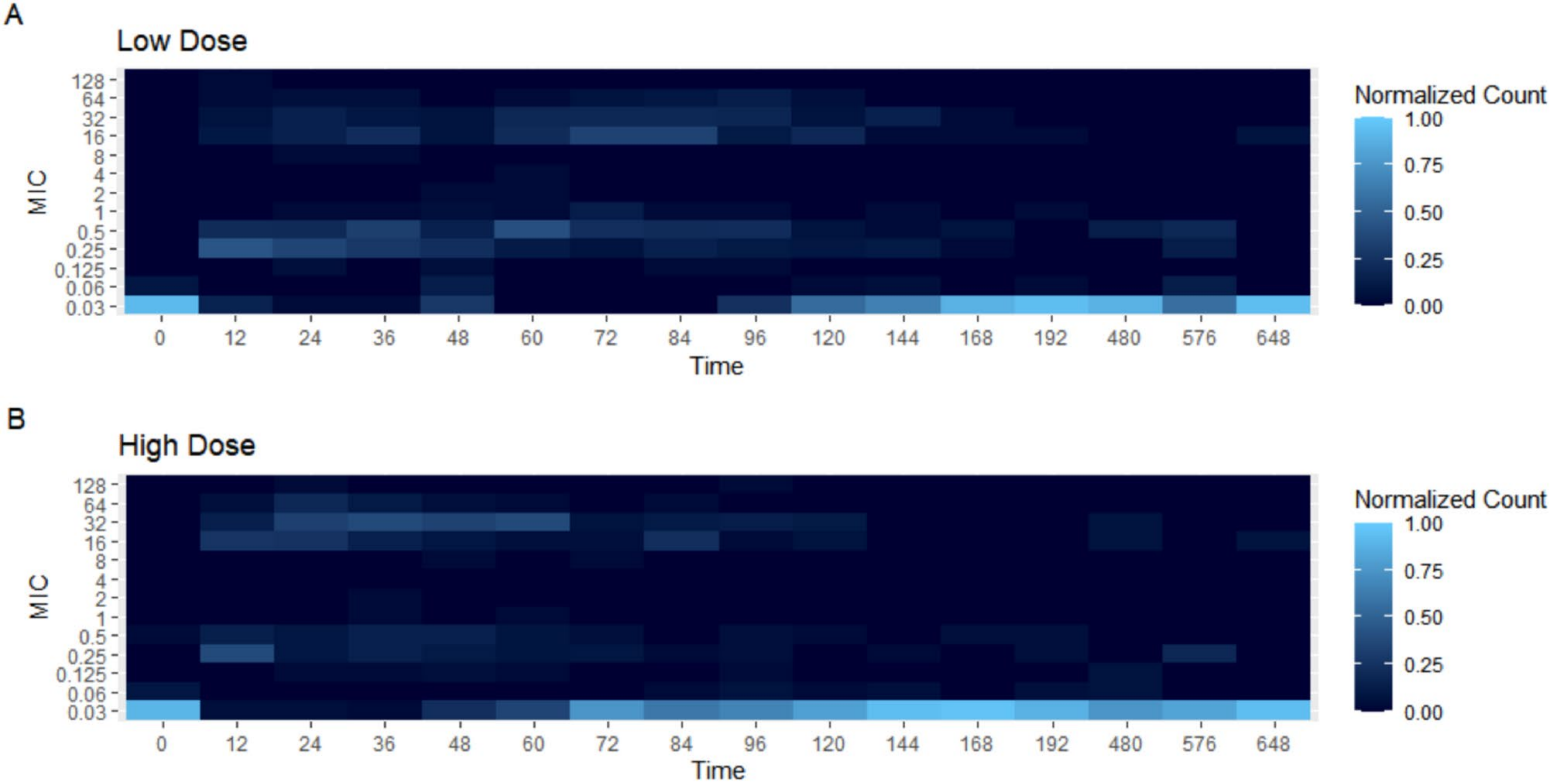
Heat Map demonstrating the number of *E. coli* isolates per each MIC value over time for both the low dose (**A**) and high dose group (**B**).

**Figure 5:**
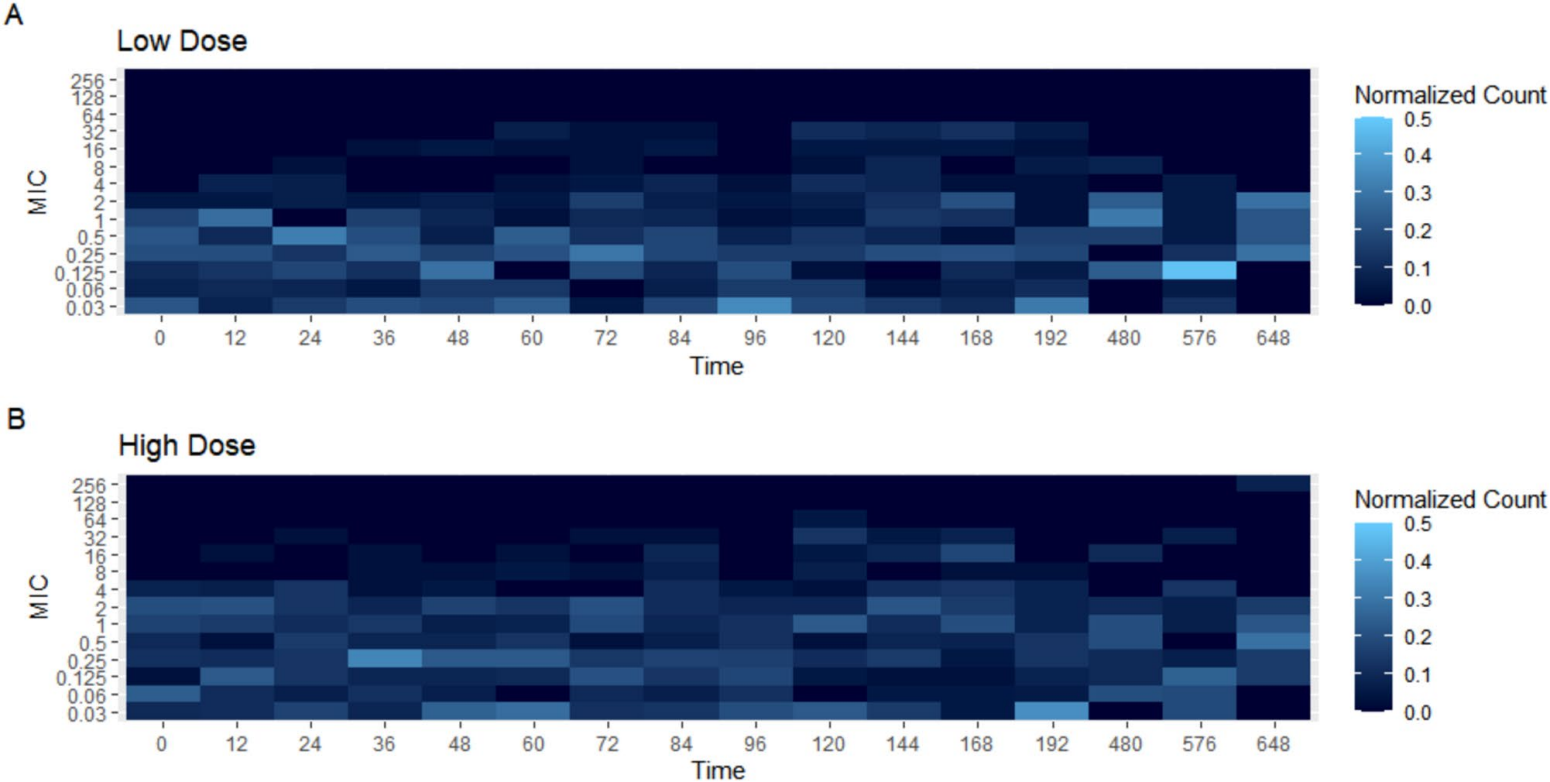
Heat Map demonstrating the number of *Enterococcus* isolates per each MIC value over time for both the low dose (**A**) and high dose group (**B**).

To better visualize the changes in MIC values per dosing group for both *E. coli* and *Enterococcus* spp., heat maps were constructed. The heat maps demonstrate the number of bacterial isolates that had a value of the designated MIC. These can be seen in Figs. 4 and 5. When looking at *E. coli*, in the high dose group (Fig. 4B), there are an increased number of isolates with MIC values ranging from 16 to 64 µg/ml over the 12 h to 72 h period. For the low dose group (Fig. 4A), there both an increase in variability of the MIC values and increased MIC values, ranging from 16 to 128 µg/ml over a longer period of time, 12 h to 144 h when compared to the high dose group. For *Enterococcus*, the low (Fig. 5A) and high dose (Fig. 5B) groups appear to look very similar. There is much more variation in the bacterial isolates’s MIC values compared to *E. coli* isolates. The variation appears to last the entire study period.

**Figure 6:**
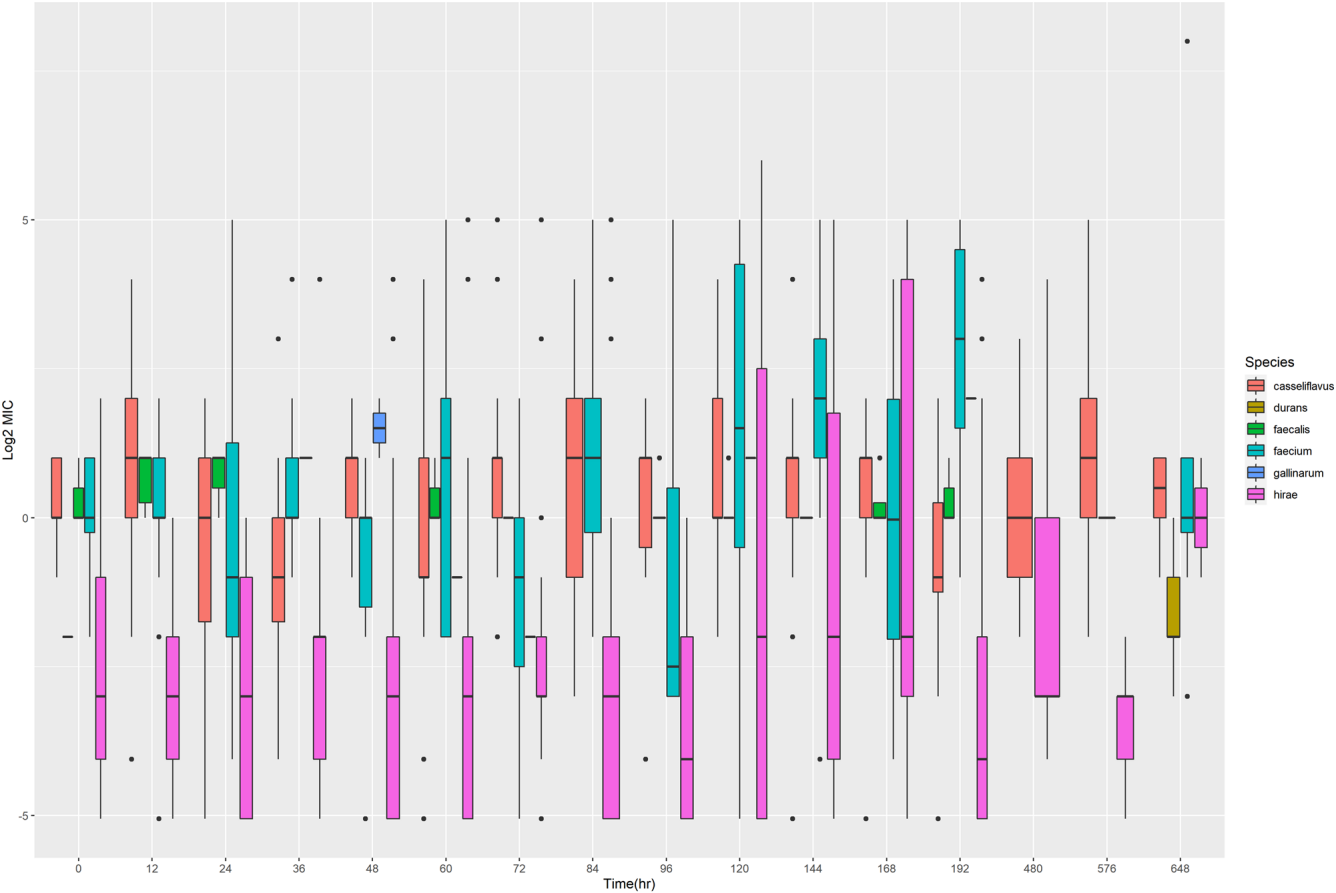
The Log2 MIC value for the identified *Enterococcus* species over time*.* The *Enterococcus* species were determined via MALDI-TOF. The data is presented as a boxplots with the upper and low quartiles present. Outliers are shown as individual black circles. Present species include *E. casseliflavus*,* E. durans*,* E. faecalis*,* E. faecium*,* E. gallinarum*,* E. hirae*.

**Figure 7:**
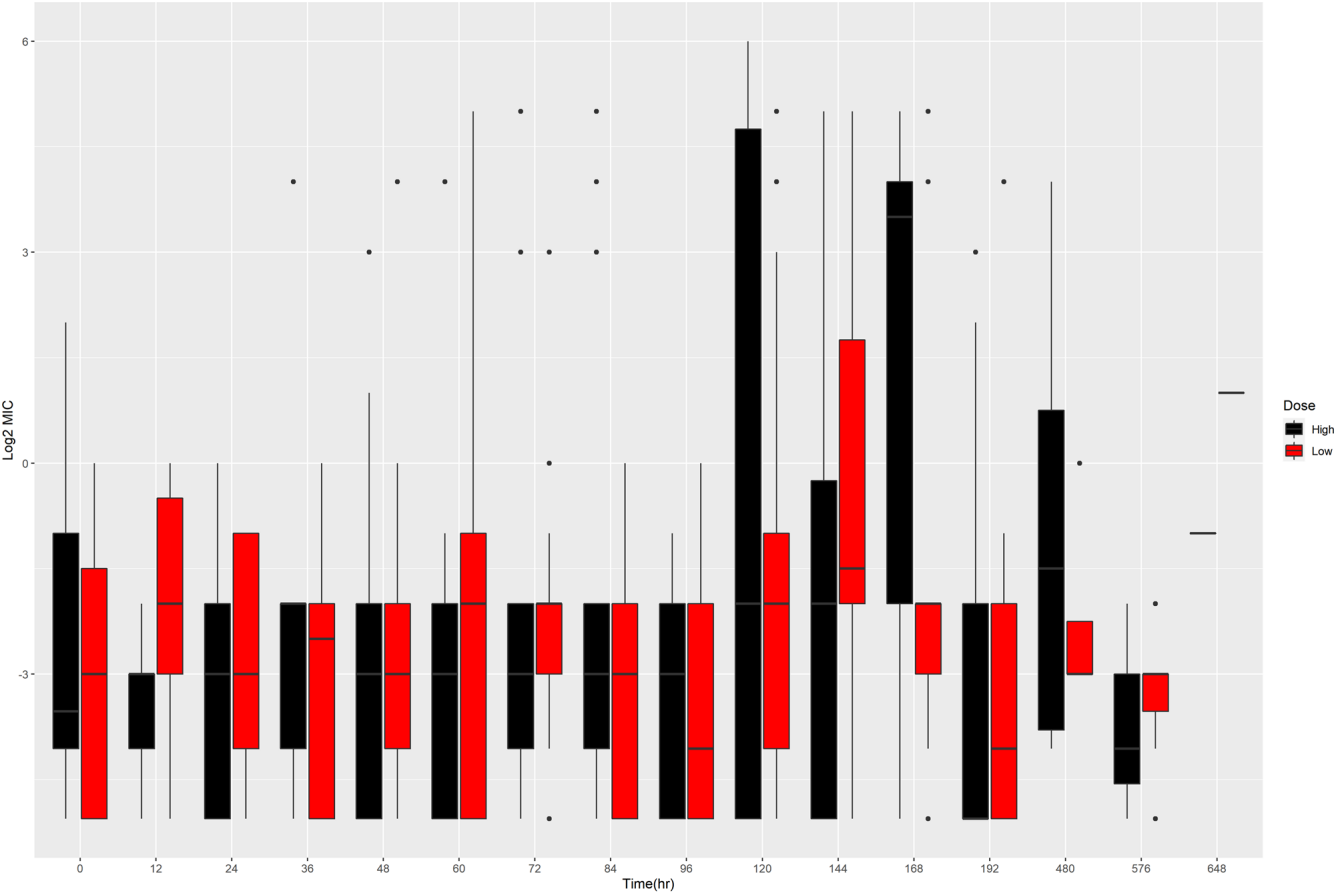
Log2 MIC over time of *Enterococcus hirae* per dosing group. The high dose group is shown in black and the low dose group is shown in red. The data is presented as boxplots with the upper and low quartiles present. Outliers are shown as individual black circles.

Individual *Enterococcus* species were evaluated individually (Fig. 6). Multiple *Enterococcus* species can be seen throughout the study period, with *Enterococcus hirae* and *Enterococcus faecium* appearing most frequently. *Enterococcus hirae*, the most commonly isolated *Enterococcus* species isolated from bovine feces can be seen in Fig. 7. Increased range of MIC values is evident in both dosing groups.

### Alterations in the fecal microbiota

**Figure 8:**
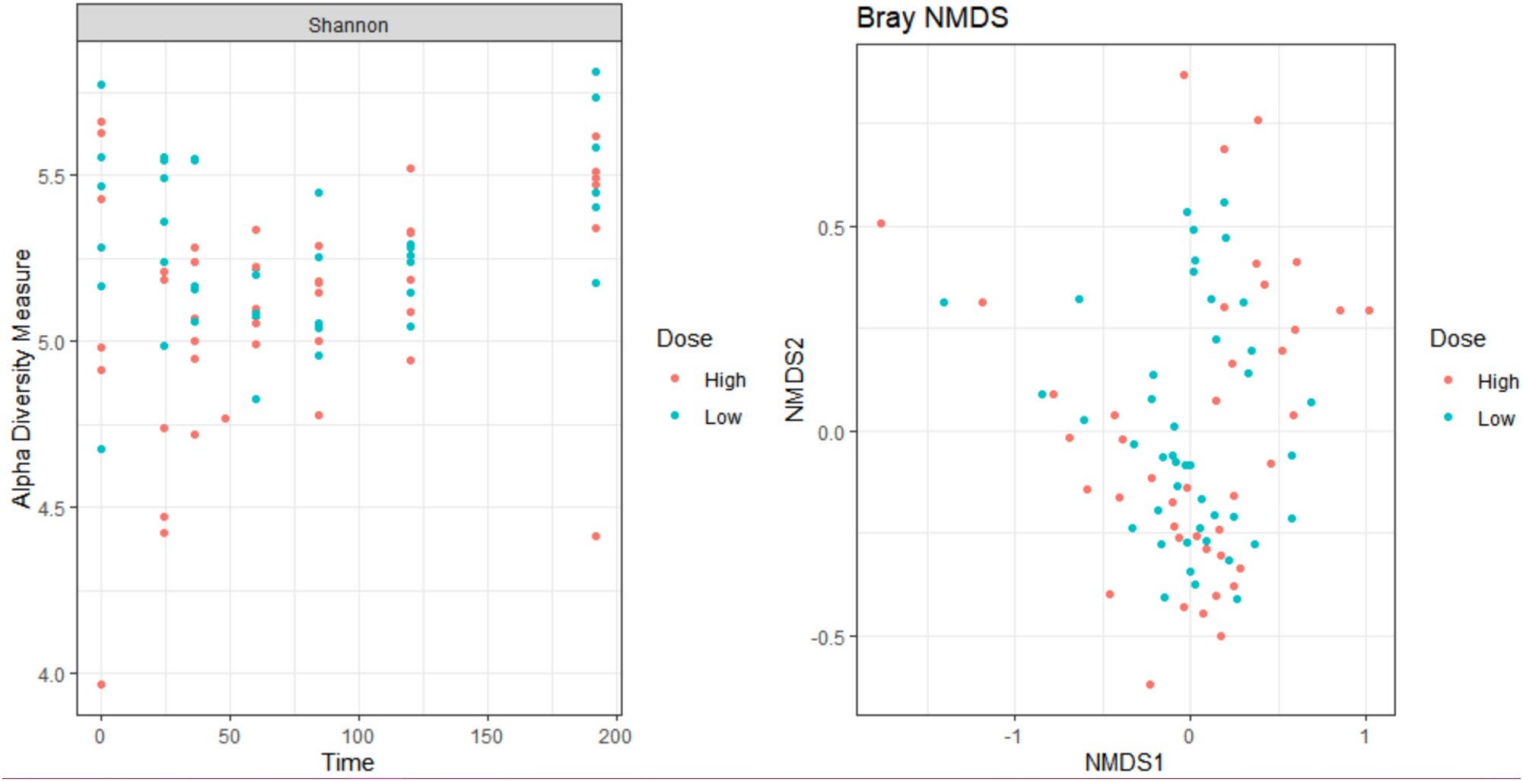
Shannon Diversity Index and Bray–Curtis plot assessing beta diversity*.* This is a Shannon Index diversity plot comparing the diversity of the samples between the high (orange) and low dose (blue) group. The higher the value on the y axis indicates there is more diversity in the microbial community. This is a Bray–Curtis plot to assess the beta diversity, or look at the variance across all the samples. The high dose group samples are shown in orange and the low dose group samples are shown in blue. The Bray–Curtis plot measures how dissimilar the samples are.

There appears to be a reduction in diversity based on Shannon Index after administration of danofloxacin, regardless of the dosing group (Fig. 8). However, it appears to rebound at the conclusion of the trial and is more similar to time point 0, before danofloxacin administration. When assessing beta diversity, the samples had similar compositions (Fig. 8).

**Figure 9:**
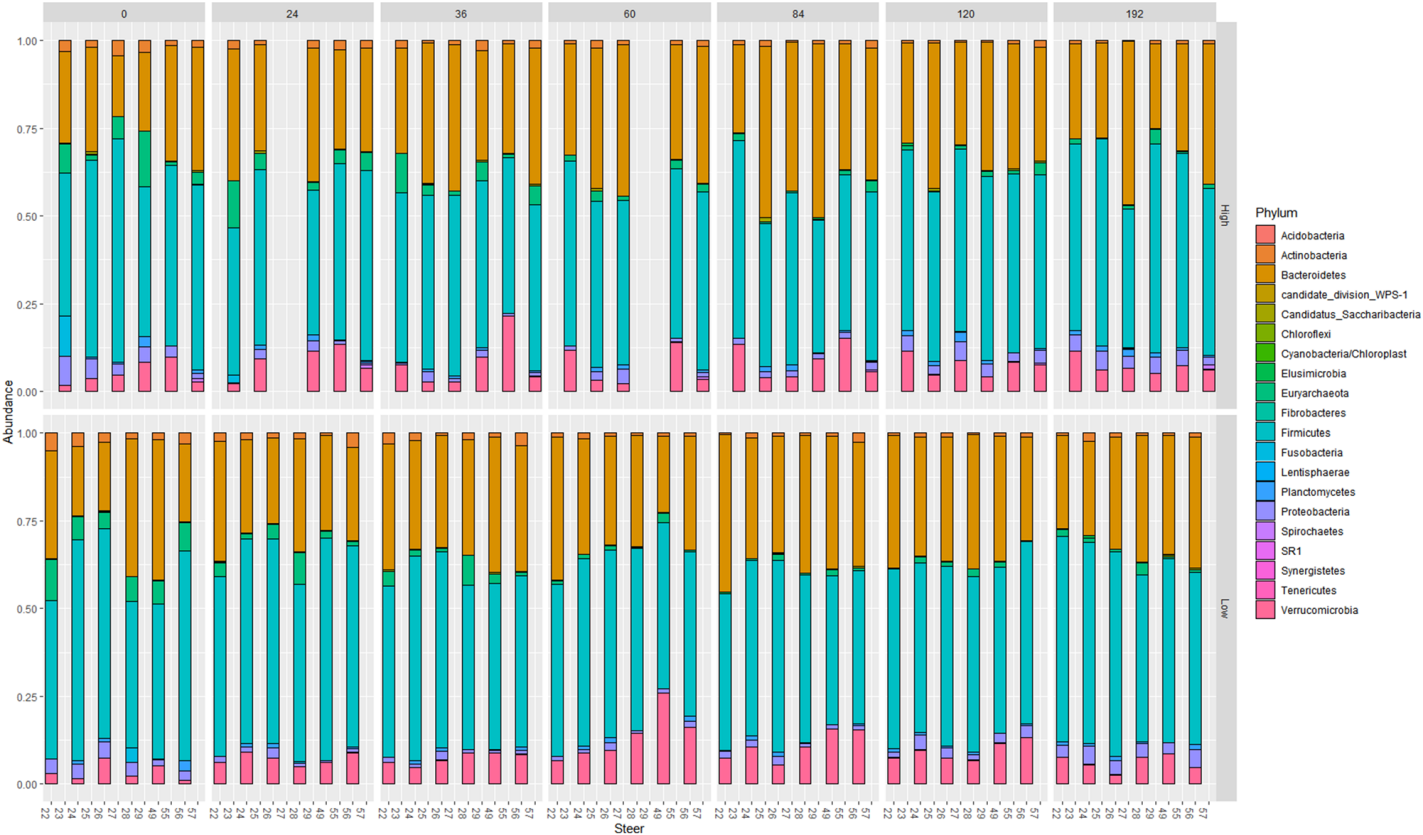
Relative abundance plotting of Phylum over time per dosing group. A relative abundance plot demonstrating the change in microbial composition (at the Phylum level) over time for each individual steer per high and low dose group.

*Euryarchaeota* appears to be decreasing over time in both dosing groups, with no evident rebound (Fig. 9). This is due to a consistent decrease in *Methanobacteriaceae* across all animals (Supplementary Fig. S1). After 48 h, regardless of the dose, there was a decrease in *Methanobacteriaceae.* This was sustained through the trial, with a slight increase in abundance at 7 days.

## Discussion

Danofloxacin is a synthetic fluoroquinolone that is commonly used in veterinary medicine as treatment for bovine respiratory disease. The two different FDA-approved dosing regimens with a short meat withdrawal interval allow for it to be an attractive treatment option. For the single and multi-dose therapy, the meat withdrawal label is 4 days from the last dose. However, due to the growing threat of antimicrobial resistance and the critical designation of fluoroquinolones by the World Health Organization, understanding the relationship between gastrointestinal pharmacokinetics, changes in the gastrointestinal microbiome and phenotypic resistance are potentially useful to mitigate the public health threat of using danofloxacin in cattle.

In summary, the intestinal permeability was high for both dosing groups, and higher than what we observed for the intestinal pharmacokinetics of enrofloxacin^14^. The explanation for greater disposition of danofloxacin into intestinal fluids compared to enrofloxacin is undetermined. Fluoroquinolones are secreted into the intestine in higher concentrations than plasma and interstitial fluid because of either active transport, or secretion in the bile, or both. The concentrations in the intestinal fluids were collected with an ultrafiltration device that collects only unbound drug. Therefore, the concentrations reported for intestinal fluids represents unbound, microbiologically active drug concentrations. These high concentrations have profound effects on the population of bacteria in the intestine. We also observed high interstitial fluid (ISF) concentrations from both the high and low dose. This may reflect a persistence in tissues for longer than anticipated, and longer than predicted from the unbound drug concentration in plasma. The protein binding reported for two concentrations produces an unbound fraction (*fu*) of approximately 0.6. Ordinarily the unbound concentration in plasma is expected to equilibrate with the unbound concentration in tissue fluids; however, the concentration in ISF far exceeded this fraction.

The concentration of *E.* coli, as determined by calculating the log_10_CFU/g of feces appears to decrease dramatically after antibiotic administration, which is again similar to enrofloxacin^14^. Over time, the CFU/g reached a similar starting point to baseline (0 h). This rebound concentration of *E. coli* was delayed in these steers treated with danofloxacin when compared to those treated with enrofloxacin^14^. The median MIC was the same at each time point for *E. coli*, regardless of the dosing group. However, when looking at the individual values, there was a significant difference in MIC value between dosing groups at Day 4 and Day 6 for *E. coli*, with the low dose group being significantly higher at both time points. Further, a notable increase in the MIC of some isolates was seen as early as 12 h in both groups with isolates with an MIC of 32 ug/ml being found out to 144 h after initial treatment in the low dose group but only until 96 h in the high dose group.

*Enterococcus* growth is more stable over time as it does not appear to be influenced by administration of danofloxacin. When the MIC of all *Enterococcus* isolates were analyzed together, there were no significant differences between treatment groups, but significant differences were seen based on the speciation of *Enterococcus*. *Enterococcus hirae* is the most common *Enterococcus* species found in cattle^15^. Although it is not a significant pathogen in humans, its increased presence could potentially allow for sharing of AMR genes with other species that are more significant human disease, including *Enterococcus faecalis* and *Enterococcus faecium*, which are frequently implicated in nosocomial human infections. We were able to see wide ranges in MIC values for all these *Enterococcus* spp., which persisted beyond the labeled withdrawal time for danofloxacin. *Enterococcus faecium* appeared to have a wider MIC range in cattle dosed with the lower, multi-dose therapy. This was in contrast to *Enterococcus hirae*, which was found to have a wider MIC range in the higher single dose therapy. Interestingly, *Enterococcus faecalis* was only found in the high dosed group. The reason for this was not apparent.

As danofloxacin targets bovine respiratory pathogens, break points have been established only for bacteria associated with bovine respiratory disease, *Mannheimia haemolytic* and *Pasteurella multocida*^16^. In contrast, break points for *E. coli* and *Enterococcus* for danofloxacin in the gastrointestinal tract have not been established, making it impossible to classify these organisms as resistant or susceptible in our study. Nonetheless, there are many isolates found soon after treatment with MIC values well beyond the typical goal of an AUC/MIC > 100 based on our PK data^17^. Analyzing the aggregate MIC data is quite difficult since it is not normally distributed and quite variable. Because of these challenges, we felt that analyzing the median MIC values was the most appropriate. For this data, the lower dose group has significantly higher MIC values compared to the high dose group for *E. coli* at Day 4 and Day 6, the meat withdrawal times for each dosing group. The increased MIC value suggests that the *E. coli* in the low dose group have an increased tolerance to danofloxacin compared to the high dose group *E. coli*. This would concur with the inverted U paradigm of building increased tolerance, or resistance, to bacteria that are exposed to sub-therapeutic levels of antibiotic early in the course of therapy^18^. In terms of reducing antibiotic resistance, an increase in the meat withdrawal interval may be warranted allow for a decrease in the tolerance of *E. coli* to danofloxacin prior to an animal entering the food chain in order to limit the possible transmission of resistance.

Although no differences were seen for *Enterococcus* MIC when all species were analyzed together, the MIC per species was different depending upon the dosing group. As stated previously, *Enterococcus faecium* was shown to have a wider MIC range in the lower dosed group that persisted for a longer time period. This too concurs with the inverted U paradigm. However, the distribution of MIC of *Enterococcus hirae* is in contrast to the inverted U paradigm and our hypothesis, although there is a spike in a wide distribution of MIC values for the low dose group later in the time course, and both groups return to relatively low MIC values by the end of the study.

Aside from the decrease in *Methanobacteriaceae*, the microbial population remains rather constant throughout the study period, despite danofloxacin’s high intestinal penetration. The genus *Methanobrevibacter* is one of the dominant methanogens present in ruminants, comprising between 0.5 and 3% of the total bacterial population in ruminants^19,20^. Methanogens are responsible for the production of methane after fermentation occurs. After danofloxacin administration, here, we see this phylum decreases and does not rebound. The composition of methanogens present in the rumen will effect cattle feed efficiency. In this study, we were unable to determine the species level to see which methanogens were no longer present and how they would contribute to feed efficiency. Further, our data is based on fecal composition, and it is unclear if these changes are reflected in the rumen, the primary site of methane production.

A major limitation in interpreting the public health importance of this study is the inability to classify recovered bacteria as susceptible, intermediate or resistant isolates for both *E. coli* and *Enterococcus* towards danofloxacin. A long term goal is to develop a microbial withdrawal interval after administration of veterinary therapeutic drugs. A microbial withdrawal interval would allow for a duration of time to pass after administration of the therapeutic to minimize the likelihood of enteric bacteria resistant to drugs of human consequence to spread through the food chain as direct pathogens or reservoirs of resistance. Although there are still many unknown components and more questions to the development of a microbial withdrawal interval, demonstrating the impact of dosing regimens for danofloxacin on MIC values of *E. coli* and *Enterococcus* populations over time demonstrate when and if the population will return to a wildtype population in the absence of antibiotic pressure. Finally, the sequencing of 16s RNA is only used to identify different taxa of bacteria present within the same, usually to the genus level, we were not able to remark on any specific AMR genes that may have been shared with the different bacterial organisms. This may be an important area to investigate in future studies.

In conclusion, both dosing regimens have high intestinal penetration, yet the changes to the fecal microbiome are minor and transient. *E. coli* concentration decreases after administration of danofloxacin, but it returns to baseline at the end of the study period. Regardless of the dosing group, *Enterococcus* spp. appear to have a wide variation in MIC that persists well beyond the labeled withdrawal interval for danofloxacin. Though there are some expected *Enterococcus* species differences, in general the lower dose regimen leads to prolonged increases in MIC of both *E. coli* and *Enterococcus* spp. when compared to the single, high-dose regimen. This will need to be taken into consideration for the development of a microbial withdrawal interval if this approach is to be successful in mitigating resistance associated with use of danofloxacin in cattle.

## Materials and methods

### Animals, surgical procedure and treatment

This study was approved by North Carolina State University’s Intuitional Animal Care and Use Committee and the care of the steers was in agreement with their policies, as well as in accordance with the guidelines and regulations from this journal (and ARRIVE guidelines). Twelve healthy 6–7 month old steers (219–310 kg) were enrolled in the study. The study size was based on previous gastrointestinal pharmacokinetic studies in order to demonstrate differences between the two dosing regimens^12,21,22^. They were judged healthy by a physical exam on presentation, with no previous documentation of any antimicrobial administration. After a 3 day period of acclimation, the steers underwent gastrointestinal surgery to facilitate placement of ultrafiltration probes, as previously described^12,21,22^. The steers were clipped and sterilely prepped on their right flank. A distal paravertebral block with lidocaine was used to provide anesthesia to the right paralumbar fossa. A linear vertical incision was made. Upon entering the abdomen, the cecum was found. Just orad to the cecum, a segment of ileum was exteriorized through the abdominal incision. A small incision was made in the ileum and the collecting loops of the ultrafiltration probe (UF-3-12, BAS; Bioanalytical Systems, West Lafayette, IN, USA) were introduced into the lumen via an introducer needle. A purse-string suture pattern was applied to secure the ultrafiltration probe. The same procedure was performed to place an ultrafiltration probe into the lumen of the spiral colon. The free ends of the ultrafiltration probes were brought out of the abdomen near the abdominal incision. The flank incision was closed in a routine manner. At the time of surgery, steers received intravenous flunixin meglumine (2 mg/kg, Banamine, Merck Animal Health, Madison, NJ, USA).

Twenty-four to 48 h after surgery, the steers were dosed with either 6 mg/kg danofloxacin (Advocin; Zoetis, Kalamazoo, MI, USA) subcutaneously every 48 h (n = 6, for a total of two doses) or a single 8 mg/kg subcutaneous dose (n = 6, a single dose). The steers were housed in pairs (one from each treatment group) and fed grass hay with supplemental grain and free access to water for the duration of the study.

### Collection of plasma

To facilitate blood collection, prior to surgery, an intravenous jugular catheter (Angiocath, Becton Dickinson Infusion Therapy Systems Inc. Sandy, UT, USA) was placed. Blood was obtained at 0, 15, 30, 45, 60 and 90 min, and 2, 4, 8, 12, 24, 36, 48 h, 48 h 15 min, 48 h 30 min, 49, 50, 52, 56, 60, 72, 84, 96, 120, 144 and 168 h. The blood was centrifuged within an hour after collection at 1000×*g* for 10 min. The plasma was transferred to cyrovials and stored at − 80 ℃ until analysis.

### Collection of interstitial fluid and intestinal ultrafiltrate

After the completion of surgery with properly placed ultrafiltration probes, the free ends of the ultrafiltration probes from the ileum and spiral colon were connected to a 3 ml evacuated tube with no additive (Vacutainer R, Becton–Dickinson, Franklin Lakes, NJ, USA). This was done by connecting the probe onto a needle of the vacuum vial needle holder at the end of the tubing. The external tubing was sutured in place along the flank. Intestinal ultrafiltrate was collected by changing the tubes at the following time points: 0, 2, 4, 6, 8, 12, 24, 26, 28, 30, 32, 36, 48, 72 and 96 h post high-dose drug administration, and additionally 50, 52, 54, 56, 60, 74, 76, 78, 80, and 84 h post low-dose drug administration The fluid was transferred into cyrovials and stored at − 80 ℃.

An ultrafiltration probe was also used to collect interstitial fluid (ISF); this probe was placed in the subcutaneous space at the withers. The free end of the probe was connected to an evacuated tube in the same fashion and suture along the back. The evacuated tube was changed at 0, 2, 4, 8, 12, 24, 36, 48, 50, 52, 56, 60, 72, 84, 96, 120, 144 and 168 h. The fluid was transferred into cyrovials and stored at − 80 ℃.

### Collection of feces

Feces was collected manually from the rectum. Time points for feces collection were 0, 12, 24, 36, 48, 60, 72, 84, 96, 120, 144, 168, 192, 336, 432, 480, 528, 576, and 648 h post first drug administration. The samples were placed into bags (Whirlpak, Nasco, Fort Atkinson, WI, USA) and stored on ice. Within an hour of collection, feces stored on ice was used for microbiological analysis. Additionally, 2 aliquots of feces were placed in cyrovials and stored at − 80 ℃ for future use.

### Drug concentration analysis

The samples were analyzed for danofloxacin using a high-pressure liquid chromatography (HPLC) with UV absorbance detection for plasma samples and fluorescence detection for tissue fluids with methods previously used for studies with enrofloxacin in calves^12,21^, but with modifications to the assay optimized for danofloxacin as described in another paper^23^. The plasma assay was performed using solid phase extraction, with 400 µl of plasma added to the extraction cartridge as described previously^12^. The ISF, colon, and ileum fluid samples were analyzed directly (without extraction) because the fluid already represented a protein-free ultrafiltrate. A validation of the assay was performed by fortifying blank plasma, or other fluids (phosphate buffered saline, HyClone, VWR, Radnor, PA, USA) with a danofloxacin analytical reference standard (Vetranal, Sigma-Aldrich, St. Louis, MO, USA) to produce concentrations for a calibration curve and quality control (QC) samples. Blank (control) samples were analyzed to measure background noise and verify that there were no interfering peaks in the chromatograms for the retention time of interest. Accuracy of the method was approximately 100% for all the spiked samples (99–101%) across the range of all concentrations. All of the spiked concentrations fell within our threshold cutoff of 15%.

### Pharmacokinetic analysis

The pharmacokinetic analysis is similar to those performed in previous studies^12,21,22^. The drug concentrations were analyzed using compartmental pharmacokinetic methods to determine the drug disposition in each calf. A computer program (Phoenix WinNonlin, V. 8.0; Certara, St. Louis, MO, USA) was used to determine the values for pharmacokinetic parameters. Plasma, ISF, and intestinal drug concentrations were plotted on linear and semi-logarithmic graphs for analysis and for visual assessment of the best model for pharmacokinetic analysis. Specific models (e.g., one, two, etc. compartments) were determined for best fit based on visual analysis for goodness of fit and by visual inspection of residual plots. The best model fit was based on the equation described in the following formula:

$$C = {A^{ - \alpha t }} + {B^{ - \beta t}} + {C^{ - K01}},$$

where C is the plasma concentration, t is time, K01 is the non-IV absorption rate, assuming first-order absorption, α is the initial steep portion of the concentration curve, and β is the slower terminal slope of the concentration curve. The analysis conducted is similar to previously described methods^12,21,22^. Secondary parameters calculated from the model included the peak concentration (CMAX), time to peak concentration (TMAX), area under the plasma-concentration vs. time profile (AUC), and the respective half-lives (t½) from each of the rate constants. The relative drug transfer from the plasma compartment to the ISF and intestinal fluids was measured by calculation of a penetration factor. The penetration factor was determined by the ratio of AUC for the tissue fluid (ISF, ileum, or colon) to the AUC for plasma:

$${\text{Penetration factor }} = {\text{ AUC tissue fluid or ISF}}/{\text{AUC}}\;{\text{plasma}}.$$

Individual student t tests were used to determine significance between dosing groups for the following pharmacokinetic parameters for all fluid types (plasma, interstitial fluid and intestinal ultrafiltrate): AUC, half-life, C_MAX_, T_MAX_ and intestinal penetration.

### Protein binding

Plasma protein binding was calculated as described in previous publications^12,21^.

Plasma protein binding was performed with a micropartition device (CentrifreeMicropartition system, Amicon Millipore, Billerica, MA, USA). Aliquots of pooled calf plasma collected prior to drug administration were spiked with danofloxacin at two concentrations (0.5 and 1.0 µg/ml) to represent concentrations in the range of those measured during the study, in replicates of 3. A 1 ml sample was added to the micropartition system to obtain a protein-free ultrafiltrate for HPLC analysis. A second set of three replicates of spiked plasma at the same concentrations were processed as described previously and analyzed by HPLC for comparison. Protein binding was determined by use of the following equation:

$$\% Protein\, binding = \frac{{\left[ {Total \,concentration} \right] - \left[ {Protein\, unbound \,concentration} \right]}}{{\left[ {Total \,concentration} \right]}}.$$

### Quantification of *E. coli* and *Enterococcus* from Feces

One gram of feces was weighed and placed into either 9 ml of EC broth (EC broth, Oxoid Ltd., Basingstoke, Hampshire, England) for *E. coli* growth or 9 ml of PBS for *Enterococcus* growth. The samples were vortexed and subsequently serially diluted tenfold into sterile phosphate buffer. The diluted samples were plated in triplicate (100 ul) on selective media; *E. coli* dilutions were plated onto HardyCHROM ECC (Hardy Diagnostics, Santa Maria, CA, USA) and *Enterococcus* dilutions onto Difco Enterococcus Agar (Becton, Dickinson and Company, Sparks, MD, USA). The HardyCHROM plates were incubated for 18–24 h at 37 ℃, while the Difco plates were incubated for 48 h at 37 ℃. Dilutions that yielded colony counts of 30–300 were used; the three replicates had colony counts performed and were averaged to determine the quantity of both *E. coli* and *Enterococcus* at each time point. From the three plates that were used to determine the quantity of *E. coli* and *Enterococcus*, 8 isolates in total were randomly selected and were streaked for isolation onto Columbia agar with 5% sheep blood (Remel, Lenexa, KS, USA) and incubated overnight at 37 °C. After incubation, each suspected *E. coli* isolate was indole tested (Indole Reagent Kovacs, Remel, Lenexa, KS, USA). Each isolate was then stored in a cryogenic vial containing LB Broth (Sigma-Aldrich, St. Louis, MO, USA) supplemented with 25% glycerol (Fisher BioReagents, Fisher Scientific, Waltham, MA, USA). They were vortexed and frozen at − 80 °C as a pure growth. *Enterococcus* isolates were speciated using MALDI-TOF mass spectrometry and then stored as described above.

### Determining the minimum inhibitory concentration

The minimum inhibitory concentration (MIC) of each *E. coli* and *Enterococcus* isolate was determined utilizing broth microdilution according to Clinical and Laboratory Standards Institute guidelines^24^. Briefly, each isolate was grown overnight on blood agar; the following day, a single isolate was collected with a sterile loop and suspended in Mueller Hinton broth (BBL Mueller–Hinton II Broth, cation adjusted, Becton, Dickinson, and Company, Sparks, MD, USA) to achieve 0.5 McFarland Standard. Fifty microliters of the bacterial suspension were added into 50 ul of twofold dilutions of danofloxacin ranging from 0.03 to 64 µg/ml. If there was growth at all concentrations, the isolate was similarly grown using concentrations that ranged from 64 to 256 ug/ml (Danofloxacin VETRANAL analytical standard, SIGMA-ALDRICH, Inc. St. Louis, MO, USA). The plates were incubated overnight at 37 °C. The first well with no visible growth was determined to be the MIC.

### DNA extraction from feces

Fecal sample DNA was extracted using Qiagen MagAttract PowerMicrobiome kit DNA/RNA kit (Qiagen, catalog no. 27500-4-EP) at the University of Michigan Center for Microbial Systems. The DNA libraries were prepared for analysis as previously described by Seekatz et al.^25^.

### 16s rRNA sequencing and microbiome analysis

The V4 region of the 16s rRNA was amplified using barcoded dual index primers^26^. Polymerase chain reactions were conducted and normalized using SequalPrep Normalization Plate Kit (Thermo Fisher Scientific, catalog no. A105100). The normalized reactions were then pooled and quantified using Kapa Biosystems Library qPCR MasterMix Quantification kit (catalog no. KK4873). Samples were sequenced on the Illumina MiSeq platform using the 500 cycle MiSeq V2 Reagent Kit (catalog no. MS-102-2003).

The FASTQ sequences were analyzed using the DADA2 method^27,28^. Amplicon sequence variants (ASV) were compiled using the DADA2 tutorial (https://benjjneb.github.io/dada2/tutorial.html). Briefly, after assessing the quality of both the forward and reverse reads, the forward and reverse reads were filtered and trimmed. Error rates were determined, as well as the number of unique sequences per sample submission. The forward and reverse reads were then merged and chimeras removed. Taxonomy was assigned using the RDP training set.

### Statistical analysis

Individual Wilcoxon ranked sum tests were used to determine significance between dosing groups for the following pharmacokinetic parameters for all fluid types (plasma, interstitial fluid and intestinal ultrafiltrate): AUC, half-life, C_MAX_ and T_MAX_.

Descriptive statistics of MIC values for both *E. coli* and *Enterococcus* were determined. Predetermined individual Wilcoxon-Ranked Sum tests were conducted to assess MIC values for each organism at high dose group day 4 to day 0, low dose group day 6 to day 0, both groups compared at Day 6 and at Day 4. To construct the heat maps, the isolates per time point per dosing group were normalized to prevent any spurious data. Statistical analysis was conducted utilizing R Software^23^.

## Supplementary Information

Supplementary Table S1.

Supplementary Table S2.

Supplementary Figures.
