# Supplementary material for: Effects of danofloxacin dosing regimen on gastrointestinal pharmacokinetics and fecal microbiome in steers
Source: Sci Rep. 2021 May 27;11:11249. doi: 10.1038/s41598-021-90647-z (PMC8160337; doi:10.1038/s41598-021-90647-z)

**Effects of danofloxacin dosing regimen on gastrointestinal pharmacokinetics and fecal microbiome in steers**

J.L. Halleran*^1^, B.J. Callahan^1^, M.E. Jacob^1^, H.J. Sylvester1, T. Prange^2^ M.G. Papich^3^, D.M. Foster^1^

^*^Jennifer Halleran, jlhaller@ncsu.edu

^1^Department of Population Health and Pathobiology, College of Veterinary Medicine, NC State University, Raleigh, NC, USA

^2^Department of Clinical Sciences, College of Veterinary Medicine, NC State University, Raleigh NC, USA

^3^Deparment of Biomedical Sciences, College of Veterinary Medicine, NC State University, Raleigh, NC, USA

Supplementary Figure S1: *Abundance plot of Methanobrevibacter over time.* An abundance plot looking specifically at the abundance of *Methanobacteriaceae* over time per dosing group. Each line on the plot represents an individual steer present in the study.


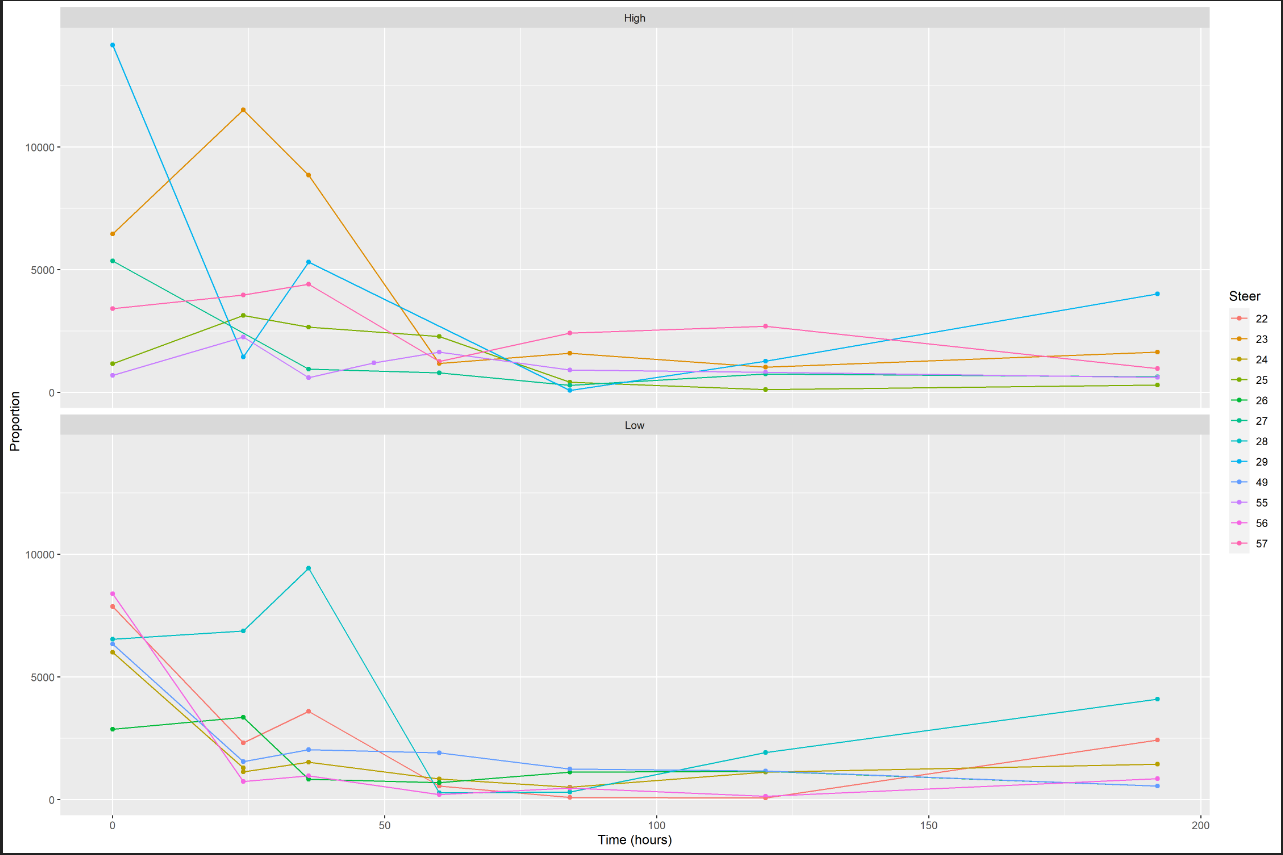

Supplement: Supplementary file 3 — Supplementary Figures. [file 41598_2021_90647_MOESM3_ESM.docx]
